# Network pharmacology and in silico approaches to uncover multitargeted mechanism of action of Zingiber zerumbet rhizomes for the treatment of idiopathic pulmonary fibrosis

**DOI:** 10.12688/f1000research.142513.2

**Published:** 2024-06-25

**Authors:** Bharath Harohalli Byregowda, Krishnaprasad Baby, Swastika Maity, Usha Yogendra Nayak, Gayathri S, Shaik Mohammad Fayaz, Yogendra Nayak

**Affiliations:** 1 Department of Pharmacology, Manipal College of Pharmaceutical Sciences, Manipal Academy of Higher Education, Manipal, Karnataka, 576104, India; 2 Department of Pharmaceutics, Manipal College of Pharmaceutical Sciences, Manipal Academy of Higher Education, Manipal, Karnataka, 576194, India; 3 Department of Biotechnology, Manipal Institute of Technology, Manipal Academy of Higher Education, Manipal, Karnataka, 576104, India

**Keywords:** Pulmonary fibrosis, Zingiber zerumbet rhizome, Network pharmacology, Molecular dynamics, Principal component analysis

## Abstract

**Background:**

Idiopathic pulmonary fibrosis (IPF) is a disease with high mortality, and there are only two specific drugs available for therapeutic management with limitations. The study aims to identify comprehensive therapeutic mechanisms of *Zingiber zerumbet* rhizomes (ZZR) to treat IPF by using network pharmacology followed battery of in silico studies.

**Methods:**

The protein-protein interaction network was developed using Cytoscape to obtain core disease targets involved in IPF and their interactive molecules of ZZR. Based on the pharmacophore properties of phytomolecules from ZZR, the drug targets in IPF were explored. Protein-protein interaction network was built in Cytoscape to screen potential targets and components of ZZR. Molecular docking and dynamics were conducted as an empirical study to investigate the mechanism explored through network pharmacology in relation to the hub targets.

**Results:**

The network analysis conferred kaempferol derivatives that had demonstrated a promising therapeutic effect on the perturbed, robust network hubs of TGF-β1, EGFR, TNF-α, MMP2 & MMP9 reported to alter the biological process of mesenchymal transition, myofibroblast proliferation, and cellular matrix deposition in pulmonary fibrosis. The phytomolecules of ZZR act on two major significant pathways, namely the TGF-β-signaling pathway and the FOXO-signaling pathway, to inhibit IPF. Confirmational molecular docking and dynamics simulation studies possessed good stability and interactions of the protein-ligand complexes by RMSD, RMSF, rGyr, SASA, and principal component analysis (PCA). Validated molecular docking and dynamics simulations provided new insight into exploring the mechanism and multi-target effect of ZZR to treat pulmonary fibrosis by restoring the alveolar phenotype through cellular networking.

**Conclusions:**

Network pharmacology and in silico studies confirm the multitargeted activity of ZZR in the treatment of IPF. Further *in vitro* and *in vivo* studies are to be conducted to validate these findings.

## 1. Introduction

Network pharmacology is a newer tool for discovering drug targets and identifying newer lead molecules to treat diseases with complex pathology. ^1^ Idiopathic pulmonary fibrosis (IPF) is one such disease where currently there is no remedy and only two specific drugs available for the treatment, nintedanib, and pirfenidone. ^2^ Nintedanib is a specific antifibrotic agent that inhibits transforming growth factor beta (TGF-β) and thereby prevents the progression of IPF. Pirfenidone has mixed antifibrotic mechanisms such as anti-inflammatory, antioxidant, and does not have specific antifibrotic activity; however, the main mechanism is found to be its antioxidant effect at the tissue level. ^3^ IPF involves genetic and epigenetic mechanisms in pathogenesis and pathophysiology. ^4^ There are multiple drug targets reported and many ongoing clinical trials, however, the priority is given in understanding the underlying molecular mechanisms and pathogenic factors would be the current focus. ^5^ Hence, there is a need to discover new drugs for IPF and the most recent trends in literature for herbal products with multitargeted approaches. ^6^^–^^8^ The network pharmacology tools are used to interpret Chinese traditional drugs for their mechanistic activity in IPF, such as Fei-Xian Formula, ^9^ Qingfei oral liquid, ^10^ Baofeikang Granules, ^11^ Astragalus Polysaccharide, ^12^ Isorhynchophylline, ^13^ and Platycodon grandiflorum. ^14^ The major pharmacological activity of these drugs is inhibiting TGF-β, multi-targeted molecular modulation of inflammation and antioxidant. We discovered many herbal drugs from traditionally used plants by literature search to screen for IPF. One such plant shortlisted was *Zingiber zerumbet* rhizome (ZZR) (Fam: Zingiberaceae) which has been reported for the ailment of multiple diseases. ^15^^–^^17^ From the standpoint of traditional use, inhabitants used Zingiber zerumbet to alleviate inflammation and respiratory diseases, including asthma and cough, in northeastern Indian states like Arunachal Pradesh, Assam, and Manipur. ^18^ ZZR possesses a broad range of bioactive compounds, and preclinical models have shown that these constituents' anti-inflammatory and antioxidant properties can affect a variety of inflammatory diseases. The ZZR contains volatile oils, flavonoids, and glycosides as pharmacologically active components. The major volatile constituent, a terpenoid reported, was zerumbone, which has multiple pharmacological activities in chronic diseases. ^19^ Taking into account the literature on ZZR that include anti-inflammatory properties that control TNF-α and IL-4 production in an inflammatory animal model. In diabetic retinopathy rats, ZZR extract administration suppressed NF-kB activation and ERK1/2 phosphorylation. This may help to prevent the regulation of the proinflammatory release cascade and fibroblast activation associated with pulmonary fibrosis. ^20^^,^^21^ ZZR prevented LPS-induced pro-inflammatory responses by NF-κB, MAPK, and PI3K-Akt signaling pathways. ^22^ It also has immunomodulatory effects in rats. ^23^ Zerumbone has been reported to inhibit the Epithelial mesenchymal transition (EMT) by acting on the TGF-β signaling pathway in epithelial A549 cells and upregulated E-cadherin levels to improve epithelial phenotype, potentially aiding in the suppression of pulmonary fibrosis. ^24^ The extract of ZZR was reported for a significant *in vitro* antioxidant activity. ^25^ Further it was reported for antioxidant property was responsible for their anticancer activity in cell line. ^26^ The active constituent of plant rhizomes are mainly terpenoids in volatile oil and many flavonoids. ^27^

Network pharmacology, systems pharmacology, and multiple bioinformatics tools can be used to evaluate the mechanistic therapy of ZZR, ^28^ and in silico drug discovery can validate the findings from network pharmacology, especially for natural products. ^29^ Several studies on IPF reported TGF-β1, EGFR, TNF-α, MMP2 and MMP9 are the major effectors in the progression of IPF, which plays a significant role in the modification of biological processes likely to be EMT, myofibroblast proliferation, and extracellular matrix (ECM) deposition. ^30^ This study outlines the mechanistic interaction of ZZR bioactive constituents with perturbation IPF system by unexplored network pharmacology studies and confirmed the reported interactions of potential molecules and identified targets by molecular docking and dynamics simulation as an evidence approach to the study findings. Molecular docking is a computer technique that examines the pattern of binding of potential components to the recognised protein binding sites and predicts the three-dimensional (3D) structure of interest with the ideal position and orientation of the protein-ligand complex. Using scoring functions, interactions, and binding affinity, the components are then ranked. In order to determine the efficiency of the ligand, MD simulation explains how atoms and molecules move over time and under specific conditions in a protein-ligand complex. ^31^^,^^32^ Following the post-simulation, principal component analysis (PCA) is used to evaluate the protein system’s conformational variations and movement correlation. Molecular interactions of multi-components with the multiple targets of fibrosis can be explored by utilizing the network pharmacological computational methods.

## 2. Methodology

### 2.1 Identification of bioactive constituents of *Zingiber zerumbet*

The active components reported in the ZZR were identified from the Traditional Chinese Medicine Systems Pharmacology (TCMSP) ^33^ ( https://tcmsp-e.com/tcmsp.php), database and by performing literature search. ^34^

### 2.2 Targets excavating

The 3D conformers of the bioactive phytomolecules were retrieved from PubChem in an SDF format. This was subsequently fed into “PharmMapper,” ^35^^,^^36^ ( http://www.lilab-ecust.cn/pharmmapper/) which predicted human targets. The predicted targets were uploaded to the UniProt database to get standard gene names and official gene symbols. ^37^ The targets involved in IPF were found utilizing the “GeneCards” ^38^ ( https://www.genecards.org/) and “DisGeNET” ^39^ ( https://www.disgenet.org/home/) databases with efficient keywords “idiopathic pulmonary fibrosis” and “lung fibrosis” for finding the differentially expressed genes in the disease condition based on the disease score. The top targets were sorted to build a network. The overlapped gene symbols, referred to as targets of zingiber zerumbet in IPF were then obtained by uploading the component gene and the disease gene using the Venny 2.1 ( https://bioinfogp.cnb.csic.es/tools/venny) duplication method mapping tool. ^40^

### 2.3 Protein-Protein interaction and identification of potential components and targets for idiopathic pulmonary fibrosis

Step 1: The target proteins of the phytomolecules in IPF were used to construct the Protein-Protein interaction networks using String app embedded in Cytoscape 3.9.1 software. ^41^ Cytoscape software served as a platform with multiple inbuilt applications, that are needed for the interaction network and analysis through the application manager. Using the string protein query, 1.7.1 ^42^^,^^43^ and the selection of the *homo sapiens* species, the targets of ZZR in IPF were imported to the Cytoscape, with the confidence score cut-off limited to a minimum of 0.40 and no further extra interactions. Each component was sorted based on the number of nodes and edges, and more than 300 edges and 40 nodes were considered for additional virtual screening.

Step 2: The hub genes were found using the Cytohubba 0.1 built-in programme. ^44^ The hub targets were built in a separate subnetwork, and the components with the highest degree, closeness centrality, and betweenness were chosen for further potential component screening. ^45^

Step 3: Screening was done with the new technique to bring the highly disease-specific targets. From the Cytoscape, by selecting a string disease query from the dropdown box and entering a specified confidence score, highly expressed disease-specific targets were imported into the Cytoscape. The top 30 hub genes based on the degree were identified by employing the analyse network tool. ^46^

Step 4: The core targets and potential components (phytomolecules) were identified by merging the top 30 disease query target and enriched targets of ZZR using a merge tool in Cytoscape. The potential phytomolecules which interact with all the identified core targets were selected for molecular docking.

### 2.4 Enrichment analysis

Enriched hub targets of 30 nodes obtained from the subnetwork and were uploaded to the database for Annotation, Visualization, and Integrated Discovery ( DAVID) database for bioinformatic analysis of KEGG (Kyoto Encyclopedia of Genes and Genome) pathway enrichment and GO (Gene Ontology) functional annotation to elucidate biological functions (biological process, molecular function, and cellular components) of targets of ZZR components in treating IPF, with significant screening criteria of P-value and enrichment score. ^47^^–^^49^ The enrichment bubble chart and bar chart were obtained from the bioinformatics tool ( https://www.bioinformatics.com.cn/).

### 2.5 Compound-Target-Pathway network

The Compound-Target-Pathway network was built by merging the Compound-Target and Pathway-Target networks in order to better understand the interactions among the phytomolecules, targets, and pathways. ^40^ Based on the pathway analysis, the top 20 key pathways were considered with the enriched hub targets to build and analyse the pathway-target network. The relationship between the primarily screened compounds with their top subnetwork targets was developed and analysed.

### 2.6 Molecular docking

*In silico* docking was carried out using the Glide module of Schrodinger software. ^50^ Alternatively, free software such as AutoDock and Gromacs could also be used. Protein information regarding the docking was considered from the UniProt with the best PDB (protein data bank) having high resolution and screening criteria.

**2.6.1 Protein and Ligand preparation**

The structural coordinates (Solved by X-Ray crystallography) for the core targets were identified and downloaded to Schrodinger Maestro from the RCSB Protein Data Bank. These proteins were prepared by a three-step process via the protein preparation wizard of Schrodinger software. ^51^^–^^53^ Alternatively, AutoDock 4 software can be used which is freely available. The imported structure was pre-processed, hetero atoms and crystallographic waters were removed from the structure, H-bond was optimized to obtain the low energy state of protein at pH 7.4 using OPLS3e force-field. ^54^^,^^55^ Using the LigPerp programme, the structures of phytomolecules retrieved previously for biological target prediction were optimised to yield 3D coordinates. ^56^ The ligands’ possible ionisation states were generated using the Epik tool at pH 7.4, generated tautomeric states for each ligand, and the output was saved in maestro format. ^57^

**2.6.2 Extra precision (XP) glide dock and binding free energy**

A site map tool was utilised to determine and evaluate the druggable pocket in proteins that did not have binding ligands. ^58^ Subsequently, the receptor grid generation tool was used to generate a receptor grid around the co-crystalized ligand by picking it from the workspace. For the proteins, without any co-crystalised ligand-receptor grid generation tool with an entry option was used to generate a grid around the site with the highest SiteScore and bearing necessary amino acid residue to modulate protein activity. To determine and rank the potential components, rigid with XP-docking was performed and presented in an XP-visualizer, where we interpret the pose viewer file in a table and 3D visualization of the pose to interpret changes in the complex internal geometry. ^50^ Pose with a better favourable interaction score when an H-bond forms, rewards of the electrostatic and hydrophobic enclosure, lipophilic chemscore, and penalties at various conditions were all taken into account while choosing an MD simulation. In rigid docking, the hydrogen bond interaction with the important amino acids was evaluated as an independent quality measure. The Prime MM-GBSA of all the docked protein-ligand complexes was performed to identify the binding-free energy. ^59^^,^^60^ Subsequently, the protein-ligand complex exhibited a good docking score, binding free energy, and XP-dock results with the interactions were considered for the molecular dynamics (MD) simulation.

**2.6.3 Molecular dynamics (MD) simulation**

MD simulations have become an effective method for identifying the crucial residues in biomolecular interaction and assessing the stability of protein-ligand complexes. In order to examine the conformational stability and steady state of the protein-ligand complexes, 100 ns MD simulations were run using the Desmond module. ^61^ Alternatively, NAMD molecular simulation along with VMD software can be used. By using the SPC (simple point charge) solvent tool, the system builder tool was employed to develop an orthorhombic-shaped simulation box around the docked ligand complex, with a buffer distance of 10 Å from any side of the complex to keep water molecules around to prevent false interference from occurring on active sites. ^62^ By adding the necessary quantity of Na ^+^ or Cl ^-^ salts, the water-solvated system was neutralised and iso-osmolarity was maintained by adding the 0.15M salt concentration. The system was relaxed and minimised to a 100 ps local energy minimum and achieved by a gradient threshold till it reaches 25 kcal/mol/Å using the steepest descent (SD) method and the limited memory Broyden-Fletcher-Goldfarb-Shanno (LBFGS) algorithm. The custom seed MD simulation was performed with the 300K temperature and 1.0315 bar pressure, using the Nose-Hoover chain thermostat and Martyna-Tobias-Klein Barostat method coupled to an isotropic style for 100 ns using NPT. ^63^^,^^64^ At the end of the MD simulation, a simulation interaction diagram (SID) was generated to analyse the MD simulation parameters like root mean square deviation (RMSD) of protein α-carbon atoms to measure the average change in the superimposition of the selected atoms at a particular frame on the first frame, and protein root mean square fluctuation (RMSF) of the ligand fragment fluctuating while binding with the protein, and the hydrogen bonding plays a crucial role in the biomolecular interactions of the protein-ligand complex, which are then followed by hydrophobic interactions and are necessary for the drug’s metabolization, specificity, and adsorption. The strong binding of molecules to proteins preserves the protein’s compactness, as assessed by the radius of gyration (rGyr), and Solvent Accessible Surface Area (SASA) was calculated to quantify the surface area of a molecule accessible by a water molecule. ^65^

**2.6.4 Principal component analysis (PCA) and Domain cross-correlation map (DCCM)**

The PCA was employed to evaluate the dynamic motion of the protein affected by the K3R and 4AFZ binding required for the biological function to calculate eigenvectors and eigenvalues. The Desmond simulation trajectory was converted to dnd format using the VMD v1.9.4 programme. ^66^ The PCA was carried out using the BioEdit R package ^67^^,^^68^ and the interaction pattern of the binding site of ligand was analysed by performing the DCCM during the overall equilibrium period. ^69^

## 3. Results

### 3.1 Bioactive constituents of *Zingiber zerumbet* rhizomes

The TCMSP and literature search yielded a list of the 39 active components that were reported in ZZR ( Table 1).

**Table 1.** Components identified in *Zingiber zerumbet* rhizomes.

Sl.No.	Components	Name
1	34DFZ	3,4-O-Diacetylafzelin
2	4AFZ	4-O-Acetylafzelin
3	LMN	Limonene
4	AMDO	Aromadendrene oxide
5	BN	Borneol
6	CPO	Caryophyllene oxide
7	CTR	Citral
8	E4OL	Eudesm-7(11)-en-4-ol
9	HP1	Humulene epoxide I
10	HP2	Humulene epoxide II
11	HP3	Humulene epoxide III
12	HML1	Humulenol I
13	HML2	Humulenol II
14	LVA	Lavandulyl acetate
15	LNL	Linalool
16	OCHE	Octylcyclohexane
17	ZBN	Zerumbone
18	ZIB	Zingiberene
19	SBN	Sabinene
20	ECP	Eucalyptol
21	CME	Camphene
22	CMR	Camphor
23	K374TME	Kaempferol-3,4,7-Otrimethylether
24	K34DME	Kaempferol-3,4-O-dimethylether
25	K3(23DARP)	Kaempferol-3-O-(2,4-di-O-acetyl-α-L-rhamnopyranoside)
26	K32AR	Kaempferol-3-O-(2″-O-acetyl) rhamnoside
27	K3ME	Kaempferol-3-O-methylether (Isokaempferide)
28	K3R	Kaempferol-3-O-rhamnoside
29	14HM	14-Hydroxy-α-Muurolene
30	3CN	3-Carene
31	4TPN	4-Terpineol
32	ACPN	α-Caryophyllene
33	AHLN	α-Humulene
34	APN	α-Pinene
35	BCPN	β-Caryophyllene
36	BMR	β-Myrcene
37	BPAD	β-Phellandrene
38	GTRP	γ-Terpinene
39	24DFZ	2,4-O-Diacetylafzelin

### 3.2 Disease targets and compound targets mining

Using the keywords “idiopathic pulmonary fibrosis” and “lung fibrosis” as input, the databases “GeneCards” and “DisGeNET” yielded a list of 6696 and 803 disease targets, respectively. Overall, 416 potential targets in IPF were identified by performing duplication analysis and combined based on the disease score from both databases with a score above 50 in GeneCards and a disease score above 400 in DisGeNET. Candidate targets were identified from Venny 2.1 by uploading disease and compound targets of PharmMapper. kaempferol derivatives like kaempferol-3-O-rhamnoside (K3R), kaempferol-3-O-methylether (K3ME), kaempferol-3,4,7-O-trimethylether (K374TME), kaempferol-3,4-O-dimethylether (K34DME), kaempferol-3-O-(2,4-di-O-acetyl-α-L-rhamnopyranoside) (K3(23DARP)), kaempferol-3-O-(2″-O-acetyl) rhamnoside (K32AR), 4-O-acetyl afzelin (4AFZ) and 3,4-O-diacetylafzelin (34DFZ) were shared more than 60 common targets as shown in Table S1 (Supplementary file 1, Extended data ^70^).

### 3.3 Protein-Protein interaction

**Figure 1.**
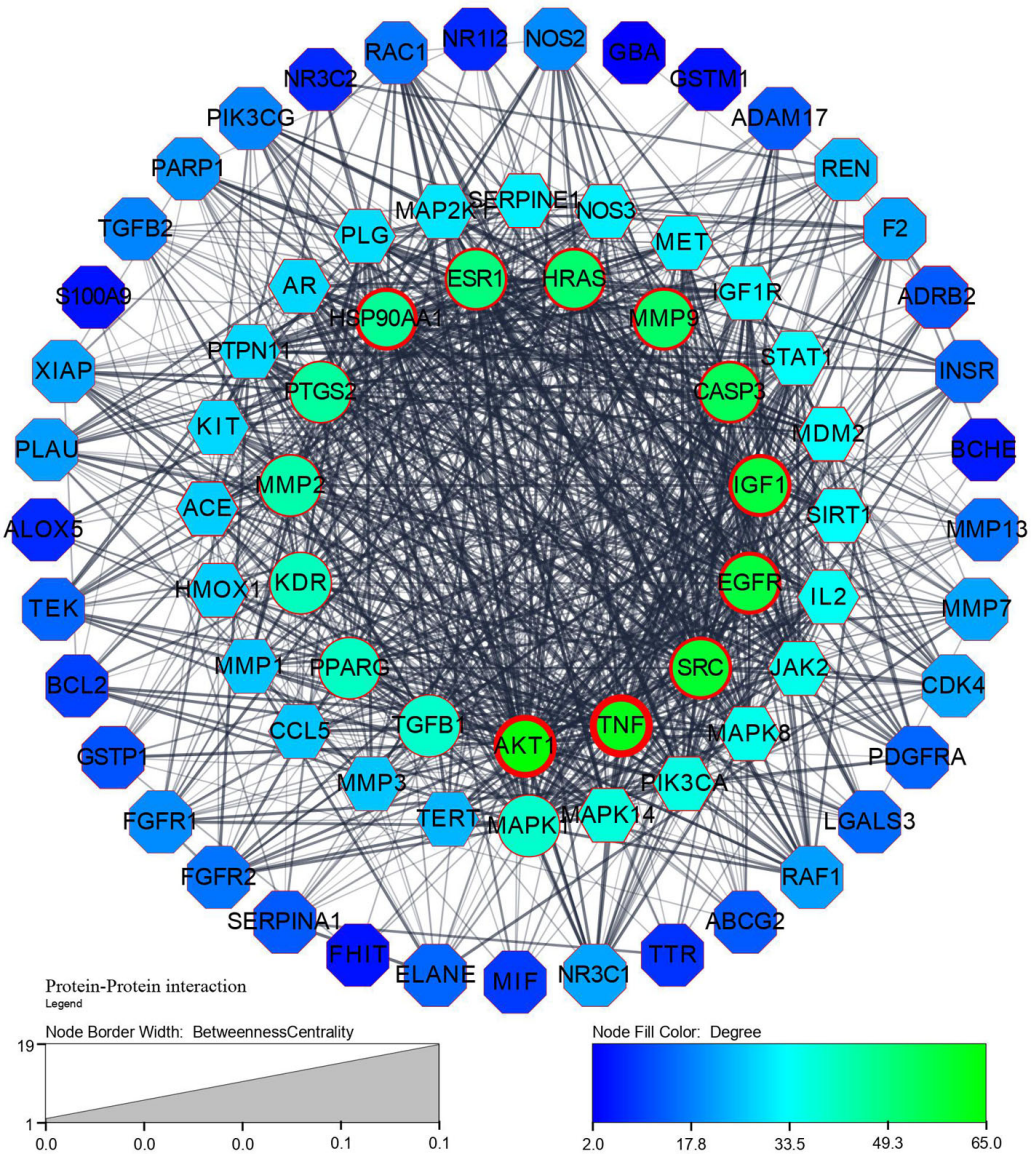
Protein-Protein interaction network and its legends of gradient colour and border width.

Step 1: A network of 76 nodes and 1010 edges was obtained for a component K3R and represented in Figure 1; similarly, networks were built for all the components and screened primarily using the criteria to obtain 25 components (Table S1, Supplementary file 1, Extended data ^70^), based on the topological parameters of networks. Targets with more than a 50-degree score are represented in gradient green colour and gradient light blue between 20-50 degrees. Targets with thick borders (more than 15) represented good betweenness centrality. A circular shape represented good closeness centrality of more than 0.666, hexagon represents closeness centrality score between 0.595 to 0.666, and octagon represented lesser than 0.595 closeness centrality.

Step 2: Table 2 lists the hub targets for all components based on the degree, betweenness centrality, and closeness centrality using Cytohubba.

**Table 2.** Enriched hub targets of 25 compounds for pathway identification and disease-specific targets identified using Cytoscape.

Enriched targets of *Zingiber zerumbet*			Hub targets of disease		
AKT1	MMP2	PIK3CA	IL18	JUN	FN1
TNF	MTOR	JAK2	IL2	IL6	IL1B
CASP3	TGFB1	IL2	MMP2	IL17A	CD44
EGFR	PPARG	HIF1A	IL10	SMAD3	TLR4
IGF1	MAPK1	ICAM1	CSF2	TP53	CAV1
MMP9	MAPK8	MDM2	FGF2	CCL4	CXCL8
PTGS2	MAPK14	IGF1R	STAT3	TGFB1	EGFR
HRAS	SRC	JUN	MMP9	VEGFA	RHOA
ESR1	CTNNB1	LNL	SRC	TNF	CTNNB1
HSP90AA1	KDR	ACE	MAPK3	IL4	AKT1

Step 3: To identify significant targets, the top 200 disease-specific targets for IPF (DOID: 0050156) were imported into the Cytoscape as a new screening method with a minimum confidence score of 0.90. After examining the network, the top 30 targets with the greatest degrees were chosen ( Table 2).

**Figure 2.**
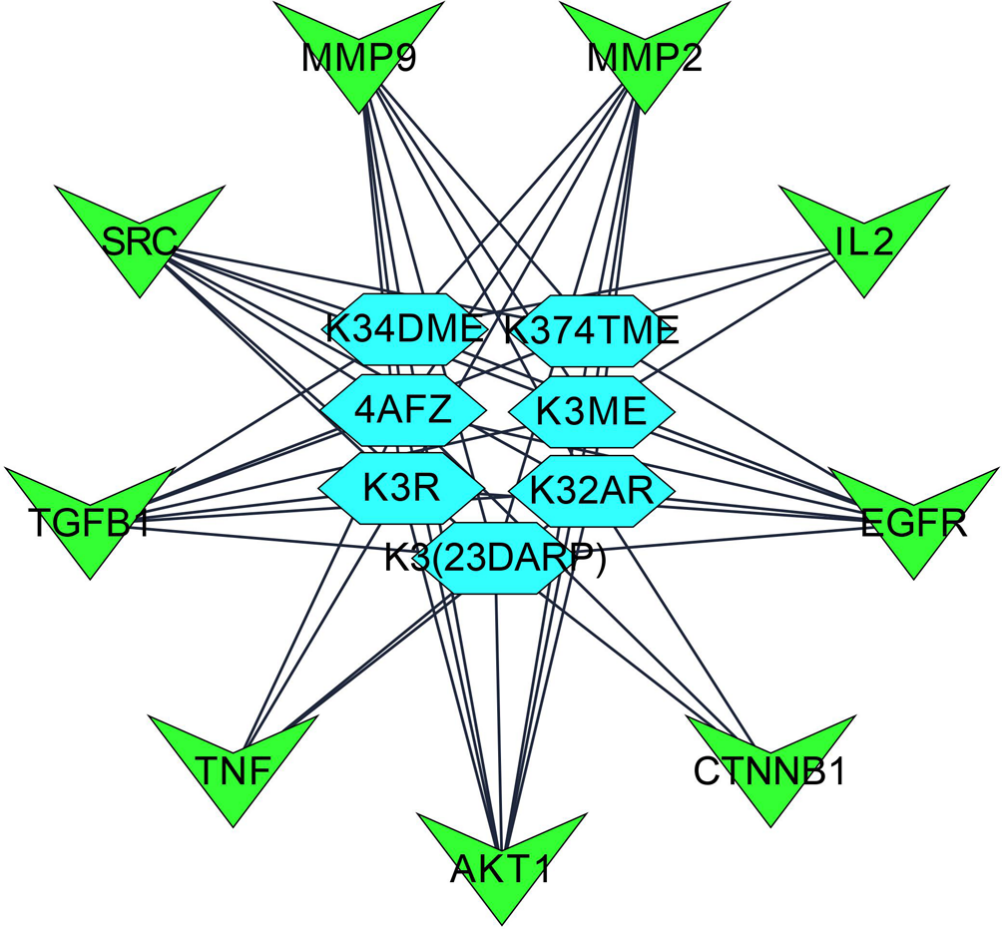
Final potential components (hexagon) and core targets network (V shape), constructed after the screening of disease-specific targets.

Step 4: Overall, seven significant components were identified with a minimum of six nodes and six edges after merging the disease-specific network and enriched targets of ZZR for molecular docking, these components with common targets were considered. According to Table S2 (Supplementary file 1, Extended data ^70^) and Figure 2, compounds such as K3R, K3ME, K374TME, K34DME, K3(23DARP), K32AR, and 4AFZ have shared common targets of TGFB1, MMP9, MMP2, EGFR, AKT1, SRC, and TNF.

### 3.4 Pathway enrichment analysis and Gene ontology functional annotation

**3.4.1 Pathway enrichment**

**Figure 3.**
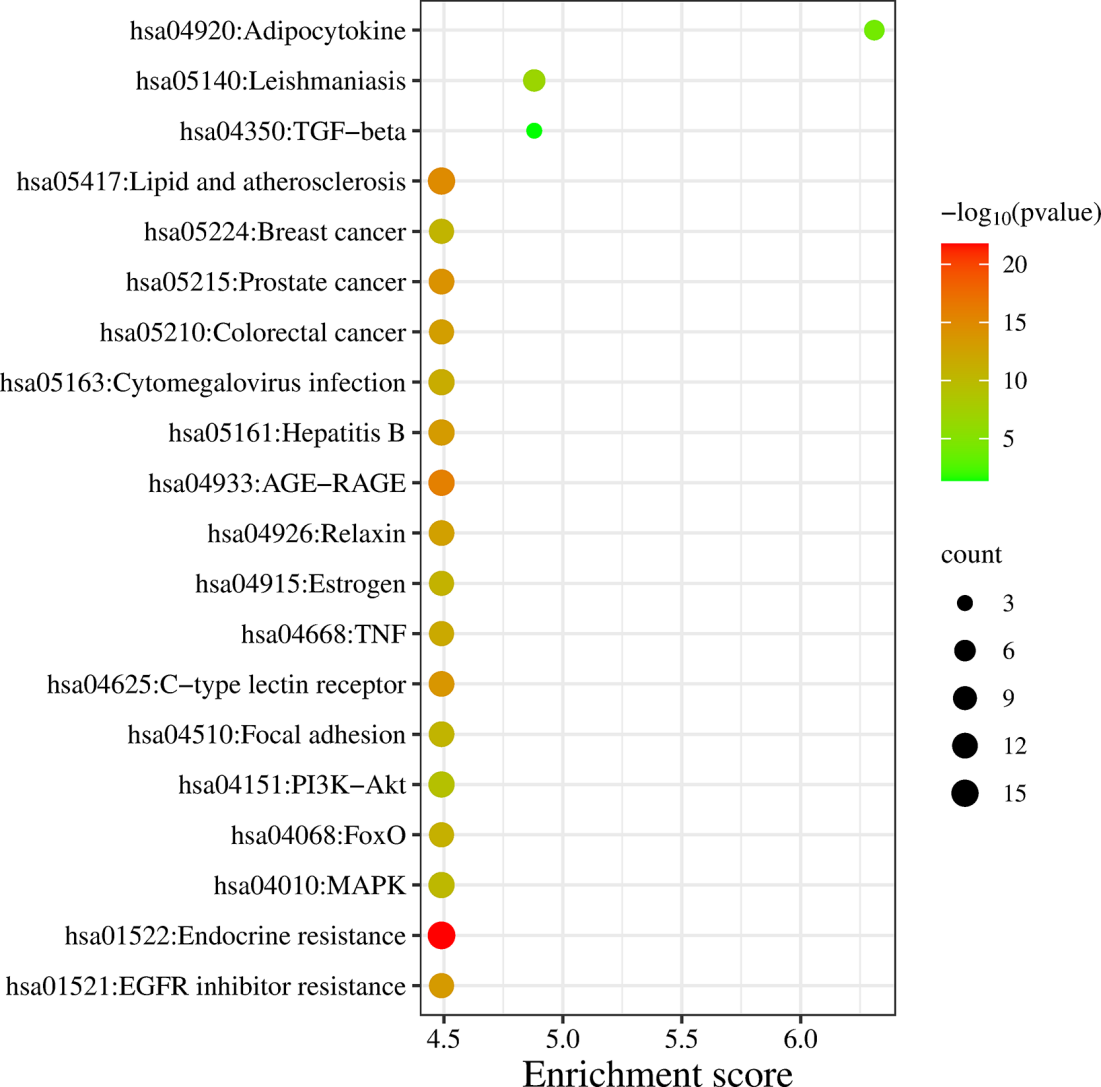
KEGG enrichment analysis of target genes.

**Figure 4.**
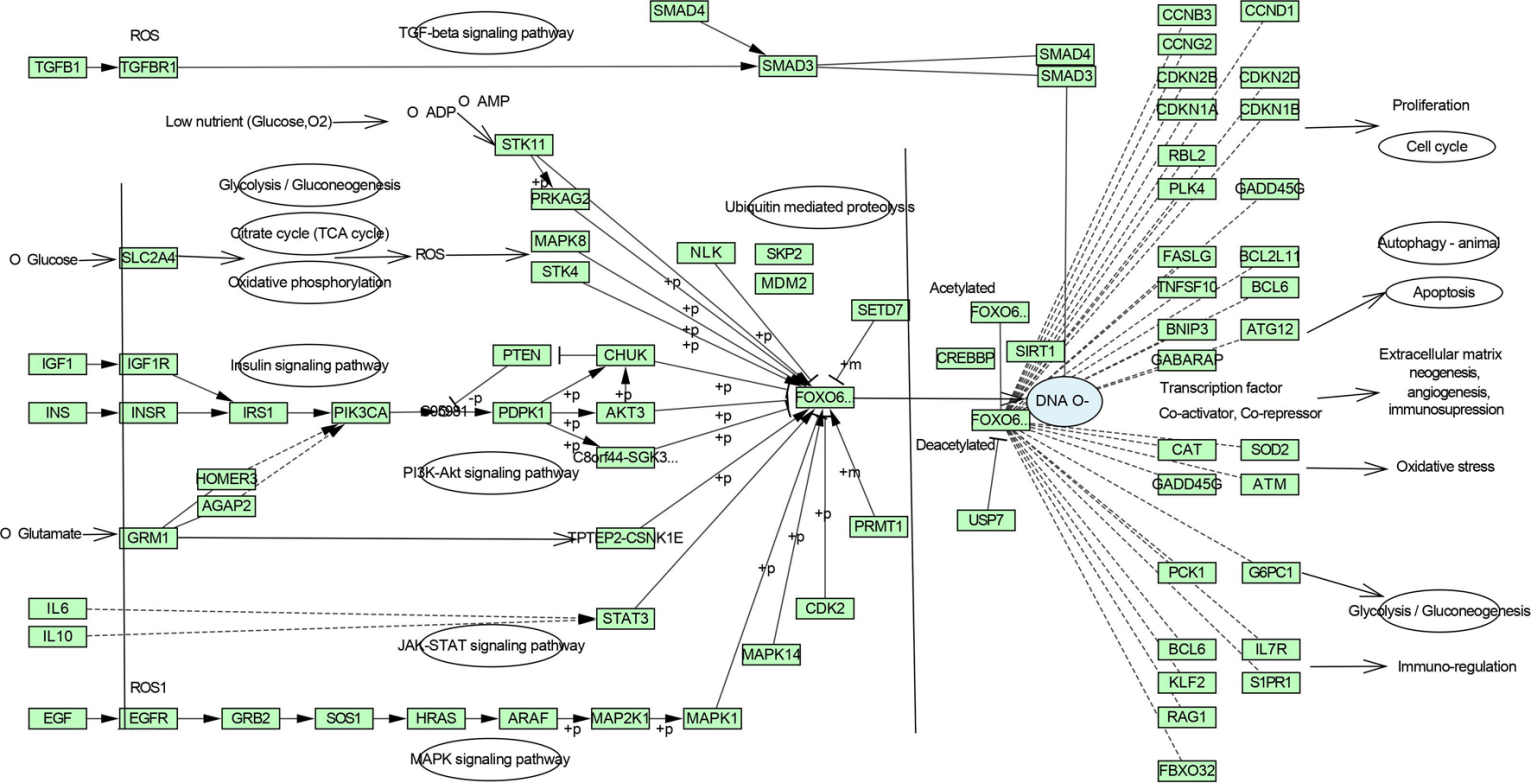
Zingiber zerumbet inhibits FoxO signalling pathway which is affected by the TGF-β and other enriched signalling pathways.

DAVID database was the keen gene ontology (GO) and pathway enrichment analysis tool with a significant P-value <0.05. The KEGG pathway was enriched by performing the enrichment analysis. Targets are enriched in 147 pathways, and the top 20 entries were considered based on enrichment score (Table S3, Supplementary file 1, Extended data ^70^), as shown in Figure 3 and 4, which comprise the TGF-β signaling pathway, PI3K-Akt signaling pathway, MAPK signaling pathway, FoxO signaling pathway, and TNF signaling pathway in IPF (Figure S1-S5, Supplementary file 1, Extended data ^70^).

**3.4.2 Functional annotation**

**Figure 5.**
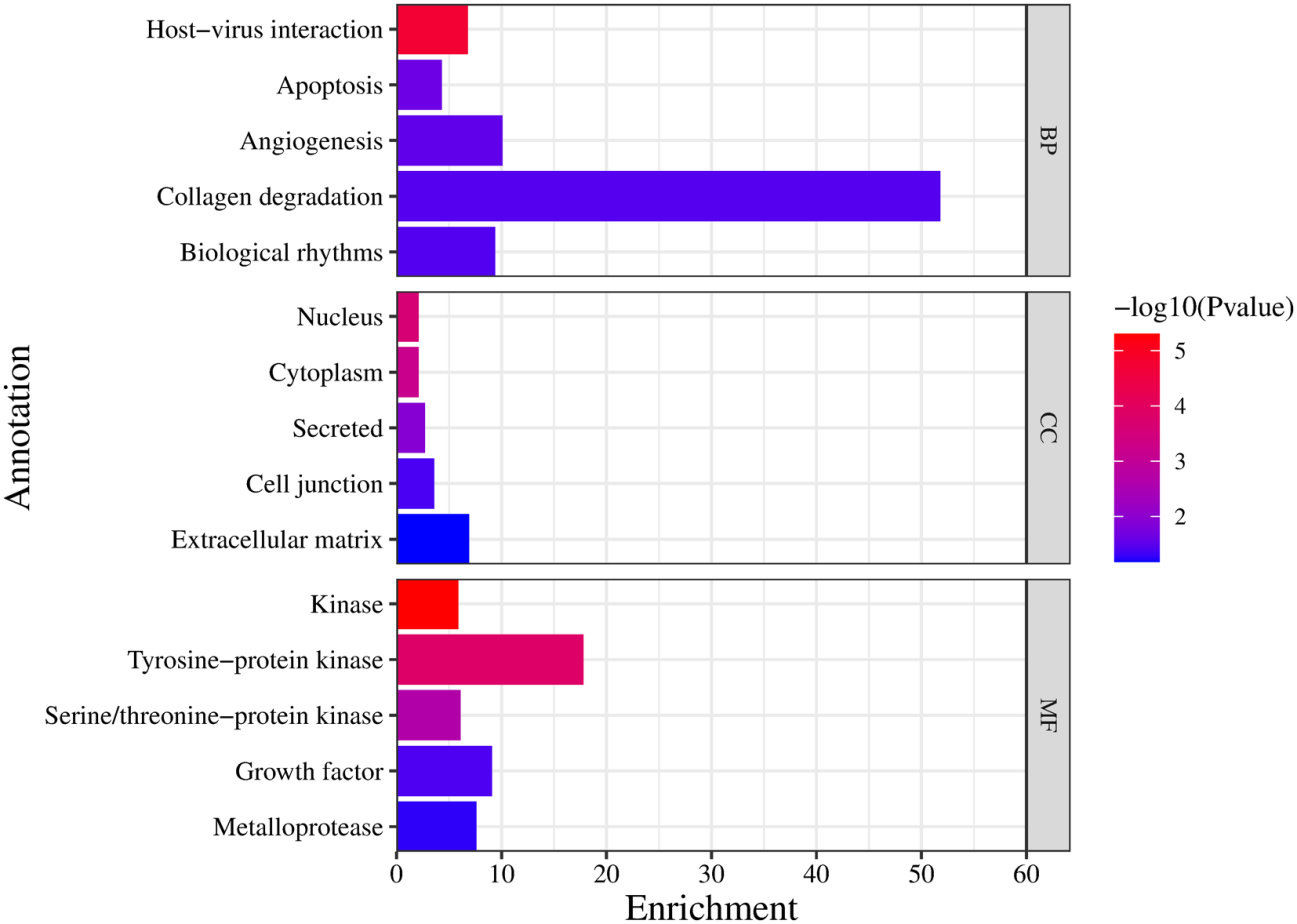
Gene ontology-functional annotation ( **BP:** Biological process **, CC:** Cellular components, and **MF**: Molecular function).

The enriched targets were uploaded to get specific functional annotations in every discipline. The biological process includes collagen degradation with the highest 51.8 enrichment scores, as represented in Figure 5 and Table S4 (Supplementary file 1, Extended data ^70^). Among the cellular components, the ECM was mostly investigated with a 6.9 enrichment score, and the molecular functions of the included entry targets consisted of the tyrosine-protein kinase with a 17.8 enrichment score.

**3.4.3 Compound-Target-Pathway network**

**Figure 6.**
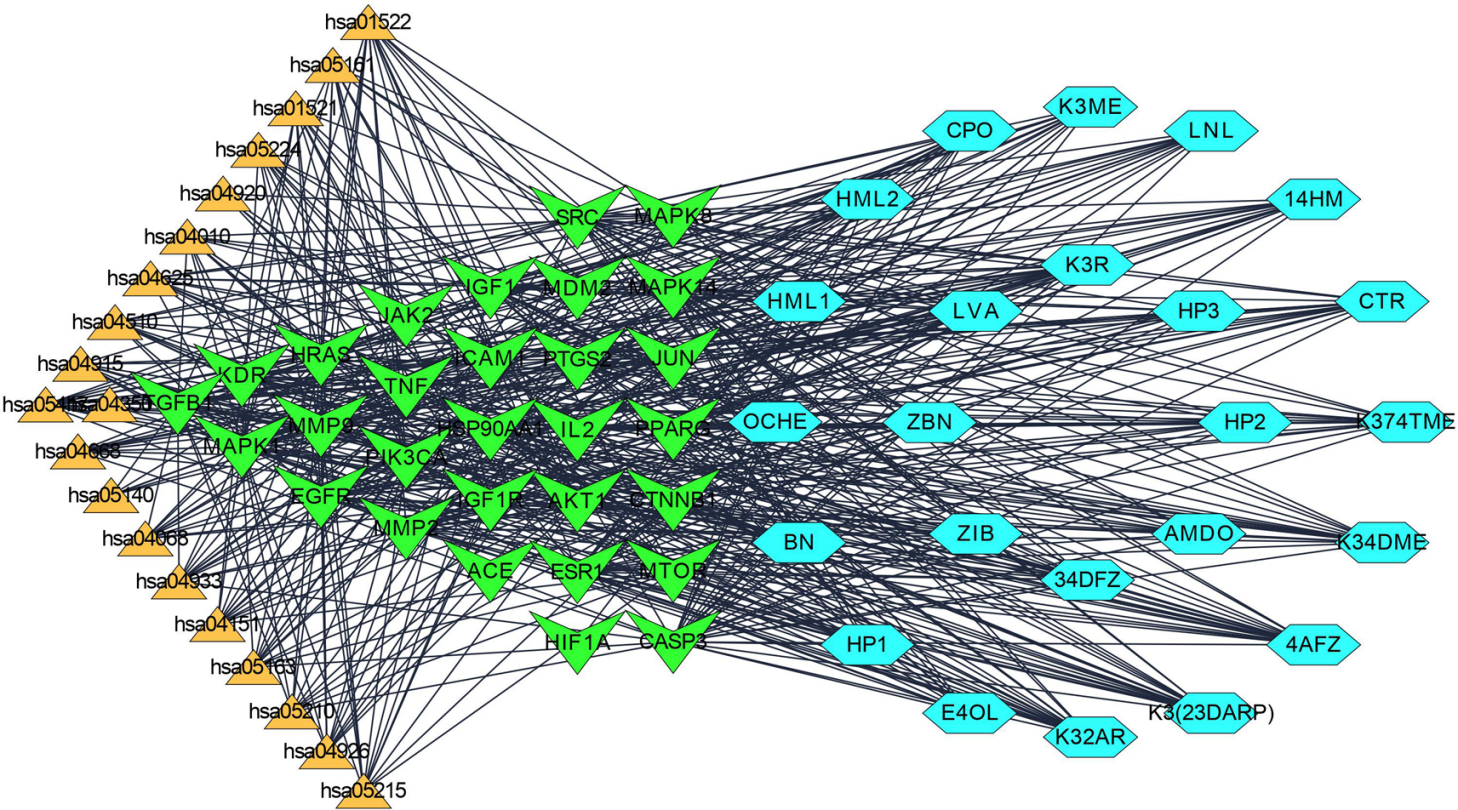
Compound (light blue-hexagon)-Target (green-V shape)-Pathway (amber-triangle) network to understand the multi-compound and target effect.

Each gene interacts with several active components, while each component targets several genes. The Compound-Target-Pathway network in Figure 6 shows compounds such as K3R, K3ME, K374TME, K34DME, K3(23DARP), K32AR, and 4AFZ interact with multiple targets. These targets disrupt the pharmacological activities of many pathways involved in the progression of IPF. The Compound-Target-Pathway (C-T-P) network was built with multi-components, multi-targets, and multi-pathways interaction, which can be correlated with a clear understanding of the pharmacological activity of ZZR. ^71^

### 3.5 Molecular docking

The identified components of ZZR were docked with 3KFD(TGFB1); 4XCT(MMP9); 3POZ(EGFR); 1QIB(MMP2); 2AZ5(TNF); 2UVM(AKT1) and 4HXJ(SRC) PDB IDs. Proteins were prepared by updating the missed side chains or loops to generate states and generated a receptor grid. The proteins without bound ligand SiteMap was used and generated a grid, with a site score of 0.976, a D-score of 1.024, and a volume of 302.18 Å ^3^, the binding site of the 3KFD was visualised and assessed as a druggable pocket at site 1. The amino acids implicated in latent TGF-β1 activation are found at this Site. The site score 0.995, D-score 0.987, and volume 395.48 Å ^3^ were found in 1QIB site 1 with the essential amino acid residues to inhibit the protein (Table S5, Supplementary file 2, Extended data ^70^).

As illustrated in Table S6 (Supplementary file 2, Extended data ^70^) and Figure S6, K3R formed a hydrogen bond with GLY 46 and ALA75 residues of TGF-β1 dimer and residues play a major role in activating latent TGF-β1. It also includes hydrophobic interaction with the reported inhibitory amino acid residues. ^72^^,^^73^ K3R interacts with ASN842 and LYS745 by hydrogen bond, and MET793 interacted by hydrophobic with EGFR. ^74^ K3R interacts hydrophobically with MMP9 amino acids of LEU188 and ALA189 residues, creating hydrogen bonds with PRO246, GLU227, and GLY186 residues. ^75^ K3R interacts with TYR119 with pi-pi interaction, SER60 with hydrogen bond and other hydrophobic interactions with LEU57, and TYR59 residues of TNF-α dimer. ^76^ Hydrogen bond interaction was noted between the K3R and GLU202 and hydrophobic interaction with LEU164, ALA165, and HIS201 residue of MMP2. ^77^ K3R exhibited higher binding free energy (∆G); -34.22, -57.35, -66.37, -48.20, and -57.85 with MMP9, TGF-β1, EGFR, TNF-α, and MMP2 respectively ( Table 3).

**Table 3.** Docking score (DS) and binding free energy(∆G)-MMGBSA (kcal/mol) of core-targets and potential-phytomolecules from *Zingiber zerumbet* rhizomes.

Compounds	TGFB1		EGFR		MMP9		TNF		MMP2		AKT1	
	DS	∆G	DS	∆G	DS	∆G	DS	∆G	DS	∆G	DS	∆G
K3R	-5.6	-57.35	-7.7	-66.37	-6.0	-34.22	-7.3	-48.20	-4.8	-57.85	-3.7	-32.71
4AFZ	-5.4	-47.79	-7.4	-79.85	-7.0	-59.52	-6.5	-45.62	-6.1	-49.94	-3.0	-37.23
K34DME	-5.0	-50.98	-6.7	-61.33	-6.1	-53.38	-6.3	-47.43	-5.5	-44.81	-4.8	-34.11
K32AR	-4.9	-57.96	-5.4	-58.25	-4.8	-47.32	-6.3	-54.44	-4.3	-17.43	-4.1	-21.97
K3ME	-4.8	-44.14	-8.3	-60.14	-5.1	-41.81	-5.7	-39.41	-4.9	-50.39	-2.6	-51.48
K374TME	-3.9	-55.96	-5.8	-61.31	-5.3	-51.51	-4.3	-33.71	-4.0	-41.59	-3.0	-19.17
K3(23DARP)	-5.1	-51.25	-5.8	-60.36	-4.4	-50.69	-5.9	-52.58	-4.2	-33.19	-3.7	-42.57

As demonstrated in Table 3 and Table S7 (Supplementary file 2, Extended data ^70^), 4AFZ provided a decent docking score and binding free energy, however specific amino acid residual interactions were only seen with the MMP9, MMP2, and TNF-α. Following glide docking, the reported 2D interactions of 4AFZ with MMP9, MMP2, and TNF-α were shown in Figure S7 (Supplementary file 2, Extended data ^70^). 4AFZ shared two hydrogen bonds with the residues GLU227 and ALA191, as well as other significant hydrophobic interactions with MMP9. ^75^^,^^78^ The MMP2 inhibitory amino acid residues of GLU202 and ALA165 have formed hydrogen bond interaction with the 4AFZ. ^77^ Ligand formed hydrogen bond interaction with SER60 and GLN61 as well as hydrophobic contacts with TYR119 residues in TNF. ^79^ 4AFZ displayed a decent binding free energy (∆G); -59.52, -45.62, and -49.94 with MMP9, TNF-α, and MMP2 respectively.

The structure of K3R and 4AFZ were illustrated in Figure S8 (Supplementary file 2, Extended data ^70^) and considered for dynamics simulation at 100 ns based on good docking score except for AKT1 and SRC, hydrogen bond interaction scores obtained from the XP-visualizer (Table S8, Supplementary file 2, Extended data ^70^), good binding free energy, and substantial interaction with the desired amino acid residues of all proteins to inhibit their activity. ^80^ The hydrogen bonds were formed within the geometric criteria of 2.5 Å in all the protein-ligand complexes as shown in Table S9 (Supplementary file 2, Extended data ^70^) except EGFR-K3R of GLY791 (2.68 Å) and MMP2-4AFZ of ALA165 (2.79 Å). The pi-pi interactions were observed within the geometric criteria of 4.5 Å between the TNF-K3R and TNF-4AFZ complexes.

### 3.6 Molecular dynamics simulation of kaempferol-3-O-rhamnoside and 4-O-acetyl afzelin

**Figure 7.**
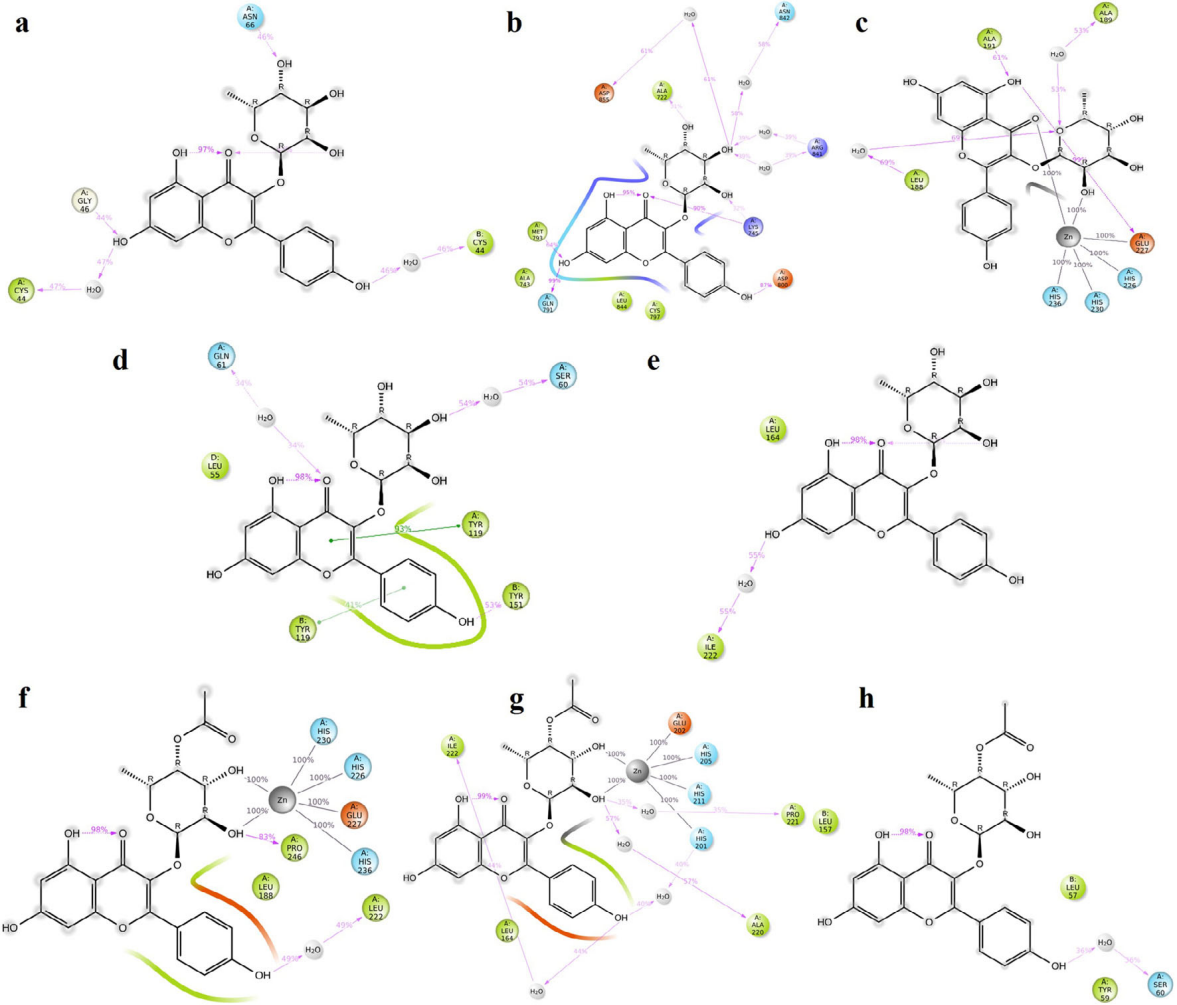
MD-2D interaction of K3R with (a) TGFB1, (b) EGFR, (c) MMP9, (d) TNF, (e) MMP2 and 4AFZ with (f) MMP9, (g) MMP2, and (h) TNF.

**Figure 8.**
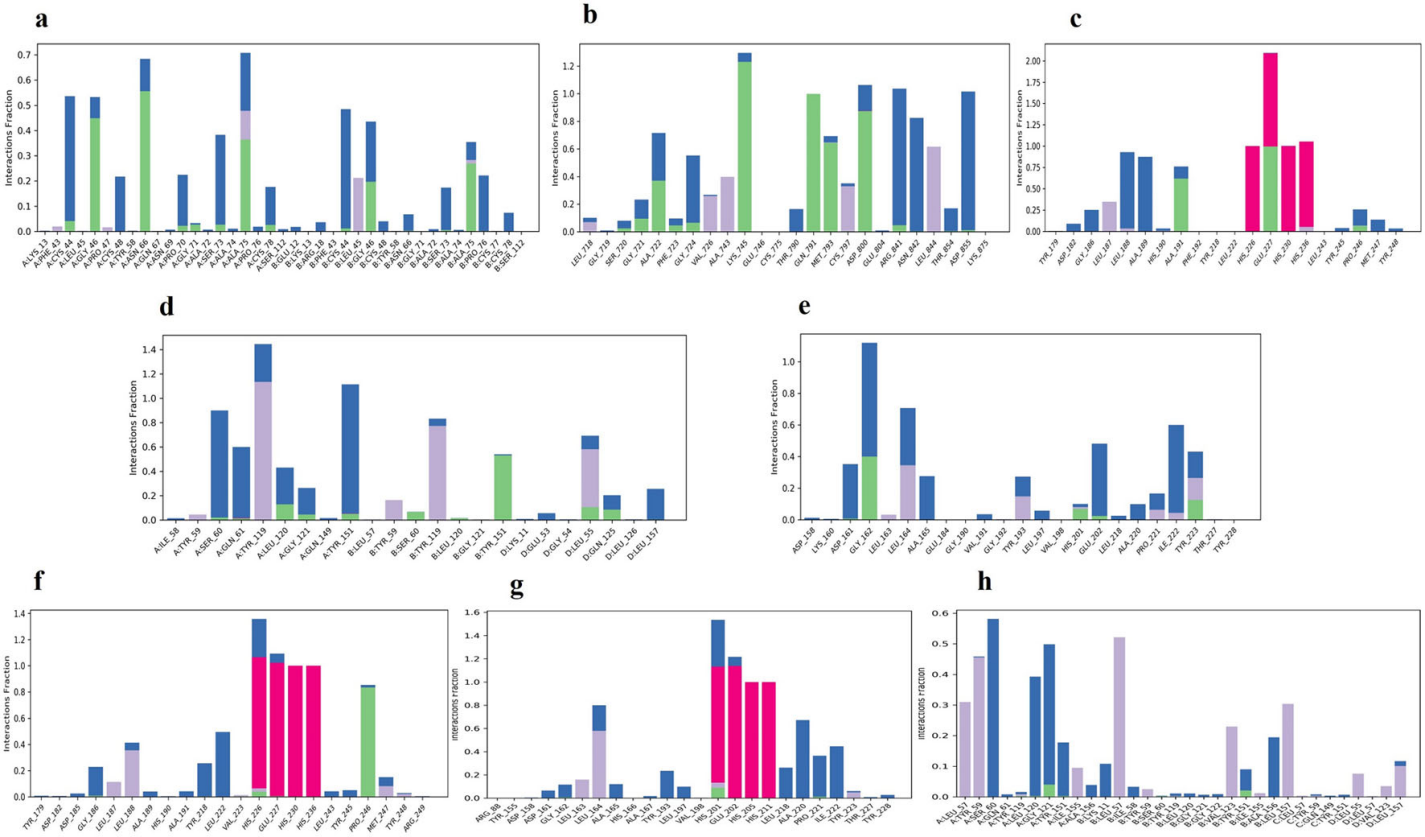
MD-contact histogram of K3R with (a) TGFB1, (b) EGFR, (c) MMP9, (d) TNF, (e) MMP2 and 4AFZ with (f) MMP9, (g) MMP2, and (h) TNF.

**Figure 9.**
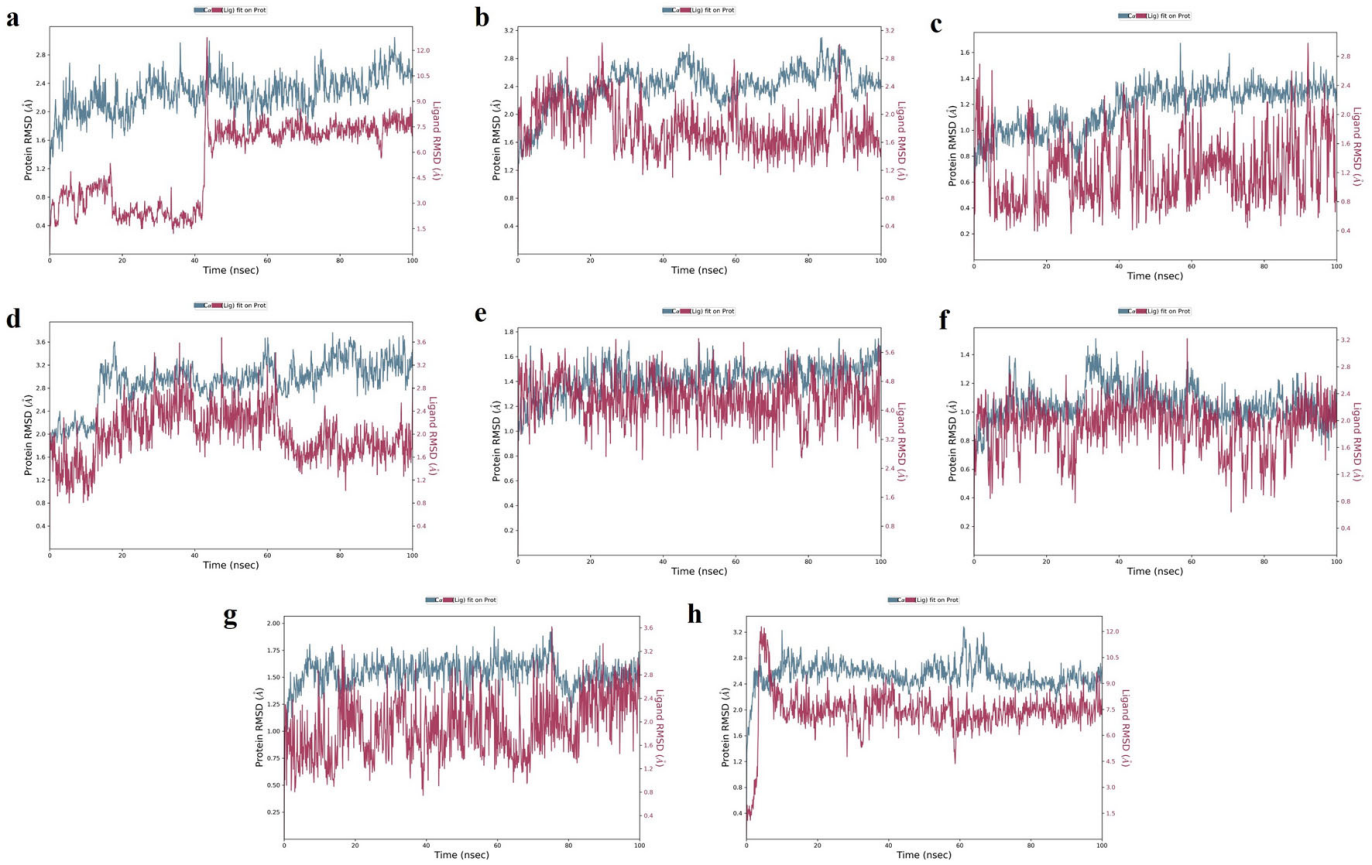
MD-RMSD of K3R with (a) TGFB1, (b) EGFR, (c) MMP9, (d) TNF, (e) MMP2 and 4AFZ with (f) MMP9, (g) MMP2, and (h) TNF.

**TGFB1:** Moderate hydrogen bond interactions were formed from the K3R to GLY46 (chain A), ASN66 (chain A), and CYS44 (chain A and B) with occupancy of 44%, 46%, and 47%, respectively, as shown in Figure 7a. ^72^ Figure 8a shows that ALA75 had hydrogen bond and hydrophobic interactions in the binding pocket. RMSD Figure 9a illustrates the stable interaction of K3R with the binding pocket of the protein within 3 Å up to 43 ns, thereafter, fluctuated for 2 ns due to conformational changes that occurred during the spatial ligand fitting and maintained continuous equilibrium till the end of the simulation period. Even during the 2 ns fluctuation, ALA75 (chain A) and CYS44 (chain B) maintained a consistent contact, but some interactions were shifted between the dimer and exposed to the solvent system. Protein RMSD is stably within 2 Å throughout the MD simulation. Inhibition of TGF-β1 suppresses mesenchymal transition, angiogenesis, ECM deposition, and myofibroblast differentiation. ^81^

**EGFR:** MD simulation retains the stability of protein and ligand interaction by succeeding to keep the molecular docking interactions within the RMSD limit. As represented in the Figures 7b & 8b, strong hydrogen bond interactions were retained in the MD simulation of K3R to residues such as LYS745, GLN791, ASP800, MET793, and ALA722 with occupancy of 90%, 99%, 87%, 64%, and 31% respectively and bridged hydrogen bonds were noted with ARG841, ASN842, and ASP855 (occupancy of 39%, 58%, and 61%). ^74^ Amino acid residues LEU844, CYS797, and ALA743 formed hydrophobic interaction, as illustrated in Figure 8b. The ligand fitting to the protein remained consistent throughout the simulation, as illustrated in Figure 9b, with 2Å RMSD being the same as protein RMSD. Inhibition of EGFR helps to decrease mucus secretion, collagen deposition, fibroblast proliferation, and disrupts lung morphogenesis. ^82^

**MMP9:** The protein and K3R interaction stabilised at the binding pocket. A strong and direct continuous hydrogen bond interaction was observed between K3R and GLU227 and ALA191 residues with 99% and 61% of occupancy ( Figures 7c and 8c). The continuous Pi-Cation (Zn) interactions were observed with HIS226, HIS230, HIS236 and GLU227. While ALA189 and LEU188 interact via bridging interactions with water molecules to K3R with occupancy of more than 50%. ^75^ From Figure 9c, the RMSD of the ligand and protein resides within 2Å. Because MMP9 is a zinc-dependent, the compound interaction required a pi-cation binding to inhibit the protein. Figures 7f and 8f illustrated 4AFZ to PRO246 formed a continuous and strongest hydrogen bond interaction at an 83% occupancy, along with the 100% Pi-Cation interactions of HIS226, GLU227, HIS230, and HIS236 via a Zn coordination, which is required for a compound’s potency to inhibit MMP9. ^75^ Figure 9f illustrates the RMSD of the 4AFZ and MMP9 within the limit of 2.5 Å and maintained the phase of stabilization throughout the period of simulation. Inhibition of MMP9 decreases the activation of latent TGF-β1 and extracellular matrix deposition. ^83^

**TNF:** From the Figures 7d and 8d, K3R displayed continuous binding stability in the binding pocket of TNF due to strong hydrogen bond interactions with TYR151 (Occupancy of 53%), moderate H-bond with SER60, and GLN61 through a bridge contact (Occupancy of 54% and 34%) and a strong 93% Pi-Pi interaction with TYR119, as well as other important hydrophobic interactions involving LEU55 and TYR59. ^76^ RMSD of ligand-protein interaction resides within 2 Å, and interaction was stabilized during the simulation ( Figure 9d).

Figures 7h and 8h show a moderate hydrogen bond formed from the 4AFZ to SER60 via a water bridge with a 36% occupancy and other key hydrophobic contacts to TYR59 and LEU57. ^76^ Figure 9h depicts the first 10 ns of fluctuation and maintains 1.5 Å RMSD for the remainder of the 90 ns simulation time to remain stable. Inhibition of TNF-α decreases inflammatory reactions and activation of macrophages. ^84^

**MMP2:** From Figure 7e, a strong hydrogen bond interaction was observed between ILE222 residues and K3R through the water bridge at an occupancy of 55%. ^77^ Hydrophobic interaction was observed in Figure 8e between the residue GLU202, GLY162, LEU164, TYR193, and TYR223 and the ligand. From Figure 9e, the RMSD was found to be within 2 Å for the Ligand and protein interaction.

Moderate water-bridged hydrogen bond interactions were formed from 4AFZ to ILE222 at 44%, ALA220 at 57%, HIS201 at 40%, and PRO221 at 35% occupancy as represented. The continuous Pi-cation interactions were seen to HIS201, GLU202, HIS205, and HIS211 through a Zn, which is required to inhibit MMP2 ( Figure 7g and 8g). The ligand fitting to the MMP2 remains consistent throughout the simulation at 2.5 Å RMSD ( Figure 9g) and maintains constant contact with the residues as depicted in the Figure 8g. ^77^ Inhibition of MMP2 suppresses β-catenin and lowers the TGF-β1 levels which favors the EMT. Inhibition promotes ECM degradation by affecting collagen degeneration. ^85^

With the exception of a few individual residues that fluctuated in the range of 40–50 and 150–160 of the EGFR–K3R complex, and 90-100 residues of the TNF-4AFZ complex, all other complexes exhibited good and lower RMSF values (Figure S13, Supplementary file 2, Extended data ^70^). The RMSF of K3R and 4AFZ ligands were stabilized at lower levels of less than 1-2 Å to their respective protein complexes (Figure S14, Supplementary file 2, Extended data ^70^), whereas the RMSF of K3R fluctuated considerably more with the TGF-β1 complex. In most of the complexes, an intramolecular hydrogen bond was observed. The rGyr of K3R and 4AFZ ranged between 4.00 ± 0.08 Å and 4.44 ± 0.10 Å respectively and maintained stability. The SASA of K3R was shown to be very less in TNF and MMP9, at 70 and 120 Å ^2^ respectively, while the other complexes with K3R and 4AFZ had an equilibrium of 240-260 Å ^2^ (Figure S15 & S16, Supplementary file 2, Extended data ^70^).

### 3.7 Principal component analysis (PCA) and Domain cross-correlation map (DCCM)

**Figure 10.**
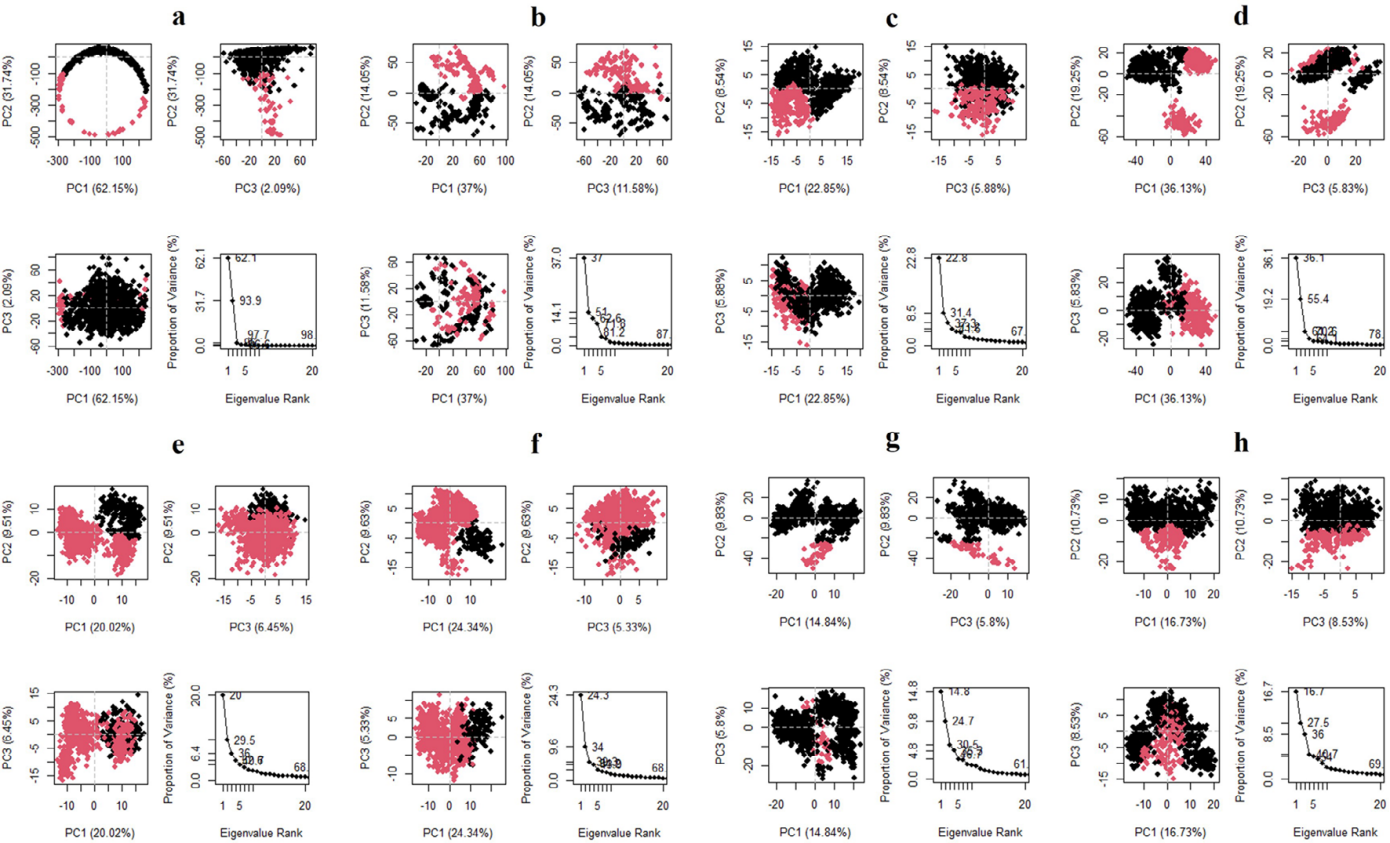
2D-PCA scatterplot, K3R with (a) TGFB1, (b) EGFR, (c) MMP9, (d) TNF, (e) MMP2 and 4AFZ with (f) MMP9, (g) TNF, and (h) MMP2.

The three principal components are represented in the Figure 10 for all the complexes of K3R with TGFB1, EGFR, MMP9, TNF, and MMP2, and 4AFZ with MMP9, TNF, and MMP2. The complex conformational variations are shown in the 2D PCA scatter plot to indicate system fluctuations by eigenvectors of PC1, PC2, and PC3. The biological functions are governed by the motion mode of 20 PCA patterns and represented in three PCA modes. The density of the distribution of the dots demonstrated stable system conformations. According to the Figure 10, except for TGFB1-K3R, PCA of all complexes exerted denser conformation, but EGFR-K3R has a small variance in the eigenvalue rank. The first three eigenvalues (PC1, PC2, and PC3) of TGFB1-K3R contributed 62.15, 31.74, and 2.09% of variables, respectively, while the other systems generated lower eigenvalues of variance ( Figure 10), indicating higher stability.

**Figure 11.**
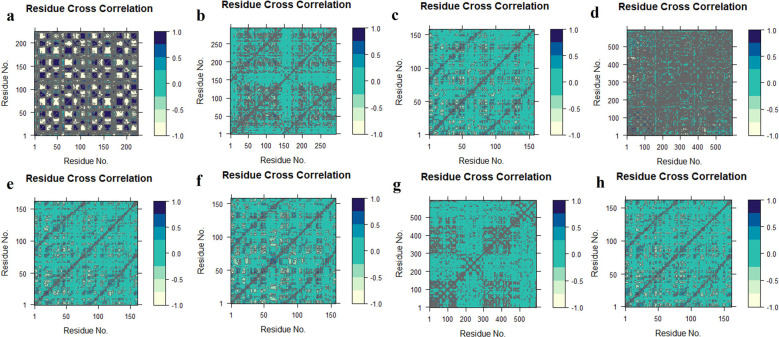
Domain cross-correlation map (DCCM) analysis, K3R with (a) TGFB1, (b) EGFR, (c) MMP9, (d) TNF, (e) MMP2 and 4AFZ with (f) MMP9, (g) TNF, and (h) MMP2.

DCCM analysis was used to examine the movement correlation of the protein complex system. When there is a positive value, the same direction emerges (positive correlated movement), however, when there is a negative value, the opposite way appears (anti-correlated movement). Except for TGFB1-K3R, the protein-ligand complex systems were shifted in the same direction as they shifted towards the cetacean blue, and dark cornflower blue implies the positive values in the Figure 11. ^69^ TGFB1-K3R correlated negatively by moving in the opposite direction towards the negative value. The colour distribution of the TNF-K3R was demonstrated to deflect the residual strength.

## 4. Discussion

IPF is a disease characterised by remodelling lung architecture due to abnormal deposition of myofibroblasts, and ECM in interstitial space by upregulation of matrix metalloproteinases (MMP2 and MMP9), resulting in respiratory failure. ^86^ As a result of a lung injury followed by an EMT brought on by TGF-β1 and differentiates myofibroblast with the growth factor receptors activation to increase the ECM deposition. ^87^^,^^88^ Chinese traditional medicines are considered over Western medicines, and many of them have been clinically shown effective in treating several diseases with a patent approach, and when treating the condition, these drugs interact with multiple pathways and targets. ^89^ In traditional medicine practices, a wide range of diseases was treated using natural products such as Chinese and Indian traditional medicine. Pertaining network pharmacology to the Indian system of medicine helps to overcome snags confronted in exploring the mechanistic effect through multi-component and multi-target by acting over a few major pathways involved in disease perturbation. ^71^ Functional analysis of hub targets by their annotation network provides a great strategy for finding the mechanism of action. Network pharmacology has prospective inferences for understating disease conditions and treatment mechanisms, and generating new avenues for investigating novel pharmacological treatments. ^90^ Here we considered rhizomes of *Zingiber zerumbet* to treat IPF with the functional hub targets, multi-component interaction analysis, confirmational molecular docking, and dynamics studies to bring forward into the new drug therapies. Network pharmacology aided in the comprehension of phytoconstituent multi-target impact, where the ‘goldilocks’ principle is followed to restore the perturbation robust network of pulmonary fibrosis. ^91^

The identified 416 disease targets from the two disease-specific databases that were screened with all components of ZZR targets and built a protein-protein interaction network. Top-25 components were found to be effective based on the higher node and edge connection as they attribute and annotate. ^92^ Targets were identified to analyse the functional annotation and pathways that interact. The course of the present study used an algorithm innovatively to screen high pulmonary fibrosis-specific targets, top disease targets were imported to Cytoscape through a string disease database and identified the overlapping component targets, and based on the degree, closeness centrality, and betweenness proximity of the network, components were selected for molecular docking. ^93^

Pathway analysis interprets, ZZR components effectively interact with the EGFR tyrosine kinase inhibitor resistance pathway to inhibit fibroblast growth, proliferation, and differentiation by acting on the epidermal growth factor receptor followed by downstream effector PI3K/AKT pathway. ^94^ MAPK signaling pathway inhibition occurs from the ZZR components to inhibit inflammation, proliferation, and differentiation-related responses through the TNF signaling pathway. ^94^ ZZR acts on the TGF-β signaling pathway by inhibiting TGF-β1 to interfere with the process of EMT, angiogenesis, ECM neogenesis, myofibroblast formation, immunosuppression, and protection from apoptosis. ^81^ The main collagen-degrading enzymes, MMP-9 and MMP-2, are likely to leave a permanent scar in lung tissue, network pharmacology and molecular docking approach to ZZR components have shown the inhibition of these matrix metalloproteinases prevent ECM deposition and scar formation. ^95^ Figure 4 interprets the inhibition of the aforementioned pathways from ZZR restrains the FoxO signaling pathway and regulates the expression of several downstream genes to improve its therapeutic effect through several aspects of the network. ^86^

The pharmacological activity of ZZR has been explained in the Compound-Target-Pathway network Figure 6 with multi-interactions. The network interactions show stable “network responses” and more effective multi-component activity than a single substance. ^96^ Illustrations of Figures 1, 2, and 6 explore TGF-β1, MMP9, MMP2, EGFR, AKT1, SRC, and TNF-α as core targets in treating IPF. The reported targets come under various stages in the development of fibrosis; inflammatory mediators release, EMT, proliferation and differentiation, and matrix accumulation. ^30^

All identified potential components exerted a good docking score of more than -4 with all core targets except AKT1 and SRC. K3R and 4AFZ had shown higher docking scores in Table 3 compared to other components K3ME, K374TME, K34DME, K3(23DARP), and K32AR. K3R and 4AFZ had specific interactions with the proteins to inhibit their activity with the hydrogen bond interactions (Table S6 and S7, Supplementary file 2, Extended data ^70^). ^80^ Based on the docking, binding free energy, and interaction analysis K3R and 4AFZ were considered for the MD simulation. Except in a few cases, the kind of hydrogen bonds formed during the molecular docking study was conserved in most of the MD simulation complexes. K3R and TGF-β1 complex conformation suffered due to the shift between the dimers. The flexibility of individual residues did not change much throughout the simulation period and preserved RMSF at lower values of complexes, except in a few cases as reported in the result section. As shown in the Figure S15 and 16 (Supplementary file 2, Extended data ^70^), the compound achieved stable RMSD, preserved structural compactness of proteins by lowering ligand rGyr fluctuation, and ligand intact in the binding pocket of protein was detected with less solvent exposure predicted from the SASA. The rigidity of the amino acids was seen in the binding pocket of the ligand-protein complexes. ^97^ Protein-ligand contacts timeline throughout the 100ns simulation period and 3D ligand-protein interactions remain stable, and data were included in Figure S9-S12 (Supplementary file 2, Extended data ^70^). K3R and 4AFZ have a potential hydrogen atom on the oxygen atom at position 28 that forms a donor hydrogen bond with the inhibitory amino acid residues of the protein targets. The donor and acceptor hydrogen bond interaction formed by the scaffold H and O atoms at positions 21, 29, 30, and 31 significantly increased the inhibitory effect of K3R and 4AFZ towards TGF-β1, EGFR, MMP9, TNF-α, and MMP2 targets in the aforementioned pathways. The connection between the TGFB1-K3R was disrupted due to opposite direction moves and varied eigenvalue. In 2D PCA scatter analysis, the coordination motion of the molecules provided lower eigenvalues of all the other complexes and demonstrated stable conformation to exert an inhibitory effect of K3R and 4AFZ to their respective complexes. DCCM examination of all other systems revealed that the good strength of the residues was achieved through positive correlation. Network pharmacology predictions had shown good therapeutic effects and scrutinized the therapeutic “multi-compound multi-target” mechanism of ZZR *,* and the activity was confirmed by molecular docking and MD simulation of K3R and 4AFZ. ^98^

## 5. Conclusion

Phytomolecules from the rhizomes of *Zingiber zerumbet* impact core targets TGF-β1, EGFR, MMP9, TNF-α, and MMP2 in IPF are expected to release immediate injury mediators, mesenchymal transition to secret ECM, fibroblast proliferation, and differentiation at various stages of idiopathic pulmonary fibrosis progression. The FoxO signaling pathway, which is connected to numerous other important pathways including PI3K/AKT, MAPK, TNF signaling pathways, and EGFR tyrosine kinase inhibitor resistance pathway, whereas the TGF-β signaling pathway has been profoundly enriched and is the pathway where phytomolecules interrupt the IPF progression along with FoxO signaling pathway. Structure-based molecular docking had emerged as a realistic approach to support and confirm the outcomes of network pharmacology with network-target by multi-component, and multi-target therapeutic understandings in the study with reliable MD simulations of K3R and 4AFZ with the core targets. Based on the proposed and confirmed affinity and stability of protein-ligand complex throughout the MD simulation period with good RMSD, RMSF, Protein-ligand contacts, rGyr, PCA, DCCM, and SASA activity of K3R and 4AFZ in ZZR is expected to better and enhance the therapeutic effect over the existing therapies to treat pulmonary fibrosis by performing preclinical and clinical studies.

## Data Availability

Figshare: Data of in silico studies on Zingiber zerumbet rhizomes for idiopathic pulmonary fibrosis. https://doi.org/10.6084/m9.figshare.24113343.v1 ^70^ This project contains the following underlying data:
- Cytoscape used to build networks of Z.z.cys - Disease Targets_idiopathic pulmonary fibrosis.xlsx - Disgenet_IPF targets.xlsx - GeneCards-IPF targets.csv - glide-dock_XP_1QIB_Zz_pv.maegz - glide-dock_XP_2AZ5_Zz_pv.maegz - glide-dock_XP_2UVM_Zz_pv.maegz - glide-dock_XP_3KFD_Zz_pv.maegz - glide-dock_XP_3POZ_Zz_pv.maegz - glide-dock_XP_4HXJ_Zz_pv.maegz - glide-dock_XP_4XCT_Zz_pv.maegz - Molecular dynamics data.zip - PCA _Output.zip - Z.z. all components targets for Cytoscape.xlsx - Z.z. David database KEGG and Functional annotation.xlsx - Z.z. sitemap result.csv Figshare: Data of in silico studies on Zingiber zerumbet rhizomes for idiopathic pulmonary fibrosis. https://doi.org/10.6084/m9.figshare.24113343.v1 ^70^ This project contains the following extended data:
- Supplementary file 1.docx - Supplementary file 2.docx Data are available under the terms of the Creative Commons Attribution 4.0 International license (CCBY 4.0)
