# Supplementary material for: Network pharmacology and in silico approaches to uncover multitargeted mechanism of action of Zingiber zerumbet rhizomes for the treatment of idiopathic pulmonary fibrosis
Source: F1000Res. 2024 Jun 25;13:216. Originally published 2024 Mar 22. [Version 2] doi: 10.12688/f1000research.142513.2 (PMC11809647; doi:10.12688/f1000research.142513.2)
Supplement: Peer review history

# Reviewer report: Reviewer response for version 2

**DOI:** 10.5256/f1000research.167734.r336034

**Published:** 2024-12-23

**Authors:** Shailendra Gurav

**Affiliations:** 1 Goa University, Taleigao, Goa, India

The manuscript presents a compelling investigation into the multitargeted mechanisms of Zingiber zerumbet rhizomes (ZZR) for treating idiopathic pulmonary fibrosis (IPF) using network pharmacology and in silico approaches.

 This approach is commendable as it integrates multiple components and targets, providing a comprehensive understanding of the treatment mechanism.

 It addresses the limitations of current IPF therapies and aligns with the growing interest in phytopharmaceuticals.

 The research focuses on IPF, a high-mortality disease, proposing ZZR as a promising alternative therapy. The study integrates network pharmacology tools, such as Cytoscape, and databases like GeneCards and DisGeNET to identify and validate core targets, including TGF-β1, EGFR, and TNF-α. Pathway enrichment analysis highlights key mechanisms like TGF-β and FOXO signalling. Molecular docking and dynamic simulations validate the stability and efficacy of ZZR components, such as kaempferol derivatives, through robust parameters like RMSD and SASA. ZZR components demonstrate multitargeted effects on pathways like TGF-β signalling, reducing fibrosis by inhibiting mesenchymal transition and extracellular matrix deposition. The study identifies kaempferol-3-O-rhamnoside and 4-O-acetyl afzelin showing strong interactions with IPF targets. ZZR’s multitargeted effects modulate key pathways, including PI3K/AKT and MAPK, addressing the multifactorial nature of IPF. This research exemplifies the power of interdisciplinary methods in advancing pharmacological discovery.

 Based on the comprehension of computational predictions, the manuscript can be accepted for indexing.

Is the work clearly and accurately presented and does it cite the current literature?

Yes

If applicable, is the statistical analysis and its interpretation appropriate?

Not applicable

Are all the source data underlying the results available to ensure full reproducibility?

Yes

Is the study design appropriate and is the work technically sound?

Yes

Are the conclusions drawn adequately supported by the results?

Yes

Are sufficient details of methods and analysis provided to allow replication by others?

Yes

Reviewer Expertise:

Natural Products Research, Computational Biology, Ayurveda Biology, Nanomedicine, Nanotechnology, Phytopharmacology, Plant Biotechnology, Phytochemistry

I confirm that I have read this submission and believe that I have an appropriate level of expertise to confirm that it is of an acceptable scientific standard.

# Reviewer report: Reviewer response for version 2

**DOI:** 10.5256/f1000research.167734.r326965

**Published:** 2024-12-18

**Authors:** Vadivelan Ramachandran

**Affiliations:** 1 JSS College of Pharmacy, Udhagamandalam, India

The author mentioned the network pharmacology and in silico  studies and the mechanism of action of Zingiber zerumbet rhizomes for the treatment of idiopathic pulmonary fibrosis.

 Validated molecular docking and dynamics simulations provided new insight into exploring the mechanism and multi-target effect of ZZR to treat pulmonary fibrosis by restoring the alveolar phenotype through cellular networking. However in the future invivo studies  to be conducted to validate further the findings

 The research article may be satisfactorily recommended for the next stage

Is the work clearly and accurately presented and does it cite the current literature?

Yes

If applicable, is the statistical analysis and its interpretation appropriate?

Yes

Are all the source data underlying the results available to ensure full reproducibility?

Yes

Is the study design appropriate and is the work technically sound?

Yes

Are the conclusions drawn adequately supported by the results?

Yes

Are sufficient details of methods and analysis provided to allow replication by others?

Yes

Reviewer Expertise:

Endocrine disorders

I confirm that I have read this submission and believe that I have an appropriate level of expertise to confirm that it is of an acceptable scientific standard.

# Reviewer report: Reviewer response for version 1

**DOI:** 10.5256/f1000research.156072.r273633

**Published:** 2024-05-29

**Authors:** Jingcheng Dong

**Affiliations:** 1 Institutes of Integrative Medicine, Fudan University, Shanghai, China

1. The identification of bioactive ingredients with mass spectrometry data is more reliable.

 2. What is the research support for selecting Zingiber zerumbet rhizome (ZZR) (Fam: Zingiberaceae) for the treatment of pulmonary fibrosis? The author lacks clinical evidence, and there is no animal experiment to support the exact curative effect.

 3. The author searched the active ingredients according to the literature, but the literature source was not shown.

 4. The pictures in Figure 3 are not fully displayed. The axis of enrichment score needs to be improved.

 5. The author should at least add animal experiments and molecular biology experiments to verify the efficacy of this drug and whether these targets of online analysis have really changed.

Is the work clearly and accurately presented and does it cite the current literature?

Partly

If applicable, is the statistical analysis and its interpretation appropriate?

Partly

Are all the source data underlying the results available to ensure full reproducibility?

Partly

Is the study design appropriate and is the work technically sound?

Partly

Are the conclusions drawn adequately supported by the results?

Partly

Are sufficient details of methods and analysis provided to allow replication by others?

Yes

Reviewer Expertise:

Network pharmacology； Chinese medicine therapy

I confirm that I have read this submission and believe that I have an appropriate level of expertise to confirm that it is of an acceptable scientific standard, however I have significant reservations, as outlined above.

# Response

**Published:** 2024-06-08

**Authors:** Yogendra Nayak

**Affiliations:** Manipal Academy of Higher Education, India

**Reviewer Comment 1: The identification of bioactive ingredients with mass spectrometry data is more reliable.**

**Answer to Comment 1:** While we agree that mass spectrometry data provides highly reliable identification of bioactive ingredients, our study utilized existing data from a well-established Traditional Chinese Medicine Systems Pharmacology (TCMSP) database and literature search. We used databases and literature based on comprehensive and validated data availability in these resources. By comparing the mass spectra of detected compounds with reference spectra from databases or published literature, TCMSP can identify known phytoconstituents present in a plant. It relies on a combination of analytical techniques and data sources to provide a holistic view of the chemical composition of medicinal plants.

**Reviewer Comment 2: What is the research support for selecting Zingiber zerumbet rhizome (ZZR) (Fam: Zingiberaceae) for the treatment of pulmonary fibrosis? The author lacks clinical evidence, and there is no animal experiment to support the exact curative effect.**

**Answer to Comment 2:** The selection of Zingiber zerumbet rhizome (ZZR) for the treatment of pulmonary fibrosis is based on its documented pharmacological properties and traditional medicinal uses. While we acknowledge the current limitations in clinical and animal studies specifically addressing pulmonary fibrosis, there is supportive data from related research areas. Traditionally, ZZR has been utilized to treat respiratory disorders such as asthma, cough, and inflammatory reactions. We have included reference articles detailing these traditional uses and have expanded the hypothesis to explore its potential in treating pulmonary fibrosis, alongside its recent applications observed in preclinical studies. In summary, the direct clinical evidence is currently lacking, the existing pharmacological data and traditional use, provided a strong rationale for further investigation.

**The following references are added:**

 18. Tushar, Basak S, Sarma GC, Rangan L. Ethnomedical uses of Zingiberaceous plants of Northeast India. *J. Ethnopharmacol*. 2010;132(1):286-96. https://doi.org/10.1016/j.jep.2010.08.032

 21. Chaung HC, Ho CT, Huang TC. Anti-hypersensitive and anti-inflammatory activities of water extract of Zingiber zerumbet (L.) Smith. *Food Agric. Immunol*, 2008;19(2), 117–129. https://doi.org/10.1080/09540100802047783

 22. Hong TY, Tzeng TF, Liou SS, Liu IM. The ethanol extract of Zingiber zerumbet rhizomes mitigates vascular lesions in the diabetic retina. *Vascul. Pharmacol*. 2016;76:18-27. https://doi.org/10.1016/j.vph.2015.08.015

**Reviewer Comment 3: The author searched the active ingredients according to the literature, but the literature source was not shown.**

**Answer to Comment 3:** We want to clarify that while we extensively searched the literature to identify active ingredients, the specific literature sources were incorporated into the methodology section. Furthermore, in addition to the literature search, we utilized the TCMSP database to identify components. We apologize for any oversight in clearly indicating the literature sources within the manuscript.

**Reviewer Comment 4: The pictures in Figure 3 are not fully displayed. The axis of enrichment score needs to be improved.**

**Answer to Comment 4:** We have addressed the issue of incomplete display in Figure 3 and improved the axis of the enrichment score. The revised figure now includes a complete image of the KEGG pathway analysis. We have utilized an enrichment score instead of an enrichment fold to denote pathways in the figure. The top three enrichment scores indicate that adipocytokines have the highest score, followed by Leishmaniasis and TGF-beta, with the third top enrichment score encompassing the remaining pathways shown in the figure. Pathways with lower scores are depicted in the supplementary file. The axis of the enrichment score was enhanced by reducing the length of the pathway names, ensuring better readability and clarity.

**Reviewer Comment 5: The author should at least add animal experiments and molecular biology experiments to verify the efficacy of this drug and whether these targets of online analysis have really changed.**

**Answer to Comment 5:** We understand the importance of in vivo and molecular biology experiments in verifying efficacy and confirming changes that may occur. However, the current study is mainly a computational prediction study. To establish a foundational understanding of the effects based on the docking studies that have shown promising results, indicating potential efficacy and target specificity, providing a strong basis for further investigation. Based on this study, we will focus on in vitro studies. These findings will be critical first steps to guide subsequent in vivo studies.
